# An episode level evaluation of the treatment journey of patients with major depressive disorder and treatment-resistant depression

**DOI:** 10.1371/journal.pone.0220763

**Published:** 2019-08-08

**Authors:** Bingcao Wu, Qian Cai, John J. Sheehan, Carmela Benson, Nancy Connolly, Larry Alphs

**Affiliations:** Janssen Scientific Affairs, LLC, Titusville, NJ, United States of America; Assistance Publique Hopitaux de Marseille, FRANCE

## Abstract

**Background:**

Many patients with major depressive disorder (MDD) fail to respond to antidepressant (AD) pharmacotherapy. The objectives of this study were to characterize MDD and treatment-resistant depression (TRD) at the level of pharmacologically treated episodes and to describe the sequential treatment patterns by lines of therapy (LOT) in the first two episodes.

**Methods:**

Adults (≥18 years of age) with continuous enrollment ≥12 months before and after the first MDD diagnosis and treated with an AD, with or without an MDD-indicated antipsychotic (AP), were identified (1/1/2010-12/31/2015). The MDD episode started on the date of MDD diagnosis that was preceded by a clean period without any MDD diagnosis. The MDD episode ended on the last MDD diagnosis or the end of the days’ supply of AD/AP medication, whichever came last. TRD was defined as an MDD episode with ≥3 AD/AP regimens. Measured outcomes included episode duration, number of LOT, relapse hospitalization, and sequential treatment patterns of MDD episode stratified by TRD and non-TRD episodes.

**Results:**

Of 48,440 patients who received AD/AP in the 1^st^ MDD episode, 3,317 (6.8%) of episodes were considered TRD. Mean duration of 1^st^ TRD episodes was 571 days, mean number of AD/AP LOTs was 3.47, and 13.7% involved relapse hospitalization. Mean duration of 1^st^ non-TRD episodes was 200 days, mean number of AD/AP LOTs was 1.21, and 9.6% involved relapse hospitalization. Among 1^st^ MDD episodes, 25.5% had a second LOT; 7.3% had a third LOT. Most patients received selective serotonin reuptake inhibitors (SSRIs) as the first LOT (63.0%), and the plurality of regimens were SSRIs in second (44.9%) and third LOT (41.1%).

**Conclusions:**

Compared to non-TRD episodes, TRD episodes were longer and more often involved relapse hospitalizations. SSRIs were the most common treatment; treatment changes and potential treatment unresponsiveness were frequent among MDD patients.

## Introduction

Major depressive disorder (MDD) is a common chronic mental disorder with 6.7% of American adults estimated to have had at least one MDD episode in 2016 [1]. It is characterized by depressed mood, persistent sadness, suicidal ideation, and frequent healthcare resource utilization. Globally, MDD is the second leading cause of disability, and it ranks second within the United States [2]. The economic burden of MDD in the U.S. is substantial; it was estimated to be greater than $200 billion in 2010, with 45% attributable to direct costs [3].

There are several pharmacologic and non-pharmacologic treatment options for patients with MDD; however, the episodic and sometimes refractory nature of MDD makes treatment difficult to manage and costly. Findings from the Sequenced Treatment Alternatives to Relieve Depression (STAR*D) trial showed that approximately one-third of patients with MDD have persistent symptoms despite receiving multiple treatments [4]. Although no consensus definition exists for treatment-resistant depression (TRD), the Agency for Healthcare Research and Quality (AHRQ) defines TRD as when a patient with MDD does not respond or remit after at least 2 trials of antidepressant (AD) treatment regimens of adequate dosage and duration, a definition consistent with Food and Drug Administration (FDA) guidance [5,6].

MDD is associated with increased physical impairment and poor quality of life. Patients with TRD generally experience greater symptom severity [7], more comorbid conditions [8,9], poorer quality of life [10,11], and higher risk of suicide [12] compared to those with non-TRD MDD. Furthermore, healthcare resource use and costs of MDD are more extensive among those with TRD [9,11–15]. At the rates of 12–20% of patients with depression, TRD is estimated to have an added annual cost ranging between $29 billion and $48 billion in the U.S. that yields higher total societal healthcare costs than those of non-TRD MDD [11].

The substantial clinical and economic burdens of MDD emphasize the need for better management, especially in the case of TRD. However, the characteristics of MDD and TRD have not been well studied at the level of treatment episodes in the real-world setting. Furthermore, the treatment patterns of MDD episodes, as well as the sequential transition through lines of therapy (LOT), are not well described. Therefore, the objectives of this study were to characterize MDD and TRD at the level of pharmacologically treated episodes and describe the sequential patterns of AD treatment, with and without an antipsychotic (AP), by LOT.

## Methods

### Data source

This analysis represents an episode-level retrospective cohort study that utilized claims data extracted from the IBM MarketScan Commercial and Medicare Supplemental databases. The Commercial database contains pharmacy and medical (inpatient and outpatient) claims of employees and their dependents; the Medicare Supplemental database profiles the health care experience of individuals with Medicare supplemental insurance. Both databases provide detailed outcomes measures, including resource utilization and associated costs for individuals covered annually by a geographically diverse group of self-insured employers and private insurance plans across the US. The patient data from the MarketScan databases are de-identified and thus in compliance with the Health Insurance Portability and Accountability Act (HIPAA).

### Patient selection

Adults ≥18 years of age with at least one MDD diagnosis (International Classification of Diseases [ICD]-9 codes: 296.2, 296.3; ICD-10 codes: F32.0—F32.5, F32.9, F33.0—F33.4, F33.9) and prescription fill of an AD, with or without a MDD-indicated antipsychotic (aripiprazole, brexpiprazole, olanzapine or quetiapine), were identified between January 1, 2010 and December 31, 2015. The date of the first MDD diagnosis was designated as the index date. All patients were required to have had continuous insurance enrollment ≥12 months prior to the index date and ≥12 months after the index date. Patients were excluded if they were diagnosed with the psychiatric comorbidities of psychosis, schizophrenia, bipolar disorder, dementia and Tourette syndrome any time during the study period. Patients included in the study were required to have ≥1 completed MDD episode during the study period, as defined below.

### Study design

**Fig 1:**
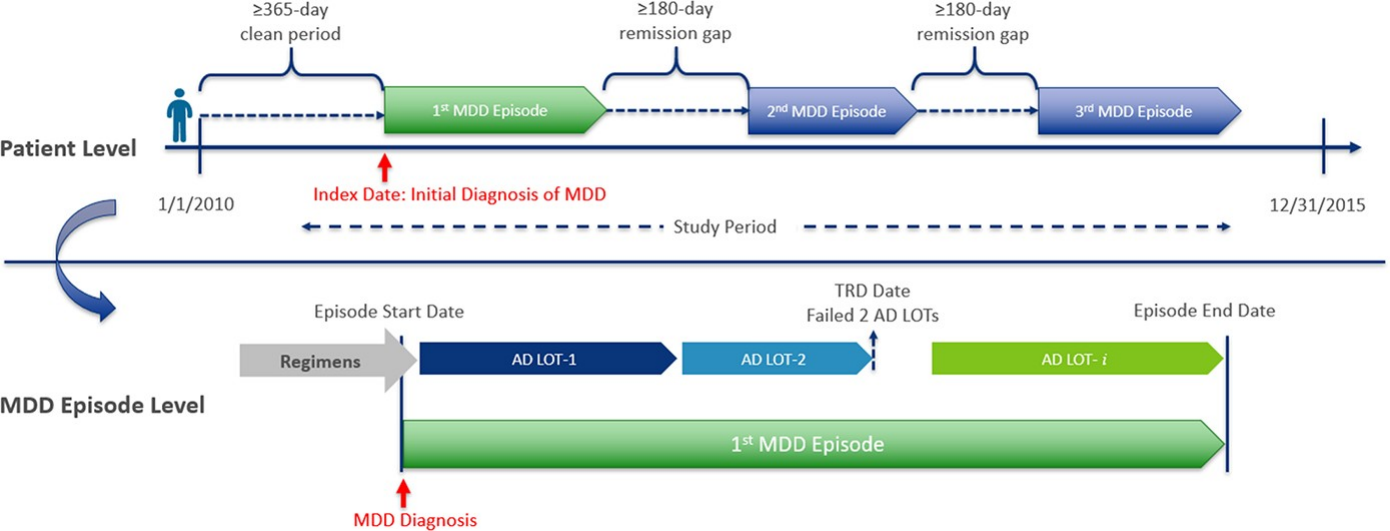
Study design. AD: antidepressant; LOT: line of therapy; MDD: major depressive disorder; TRD: treatment-resistant depression.

This episode-level analysis examined only pharmacologically treated MDD episodes, which were those when a patient had a diagnosis for MDD and a prescription fill for any AD with or without one of the MDD-indicated APs. As illustrated in Fig 1, the 1^st^ MDD episode began at the date of the first observed MDD diagnosis and was required to be preceded by a 365-day “clean period” without any MDD diagnosis or AD/AP prescription fill. A completed MDD episode was defined by ≥180 days without an MDD diagnosis or an AD/AP claim, with the episode end date assigned as the date of the last MDD diagnosis or the end of the days’ supply of AD/AP medication, whichever came last. A subsequent MDD episode started on the date of another MDD diagnosis that was preceded by a ≥180-day clean period, and the MDD diagnosis had to be accompanied by ≥1 AD/AP prescription fill during the episode. TRD was defined as an MDD episode with ≥3 AD/AP regimens, in which a regimen was defined as any combination of AD/AP used with a continuous segment of ≥28 days’ supply (allowing a maximum 60-day gap). The regimen may have included AD polypharmacy or augmentation with a MDD-indicated AP. LOTs were defined as the sequence patterns of treatment regimens within each episode.

### Study measures

Episode duration in number of days, the number of AD/AP LOT, and proportions of episodes involving a relapse hospitalization were evaluated for the 1^st^ and 2^nd^ treated MDD episodes, stratified by TRD and non-TRD episodes. Relapse was defined as a hospitalization with a primary diagnosis of MDD or suicidal ideation. For the 1^st^ treated MDD episode, patterns of AD/AP treatment regimens, as well as the treatment sequences from the first LOT (LOT1) to second LOT (LOT2) and from LOT2 to the third LOT (LOT3), were evaluated. The top 20 commonly observed treatment sequence patterns (LOT1 to LOT3) during the 1^st^ treated TRD episode were also reported. Treatment sequence patterns were described at the drug class level (i.e., selective serotonin reuptake inhibitors [SSRIs], serotonin and norepinephrine reuptake inhibitors [SNRIs], bupropion, serotonin modulators [nefazodone, trazodone, vilazodone and vortioxetine], tetracyclics, tricyclics, SSRIs + AP, and other combinations). All AD/AP included in this study were captured via General Product Identifier (GPI) codes from pharmacy claims and the Healthcare Common Procedure Coding System (HCPCS) codes from medical claims. The remission duration between 1^st^ and 2^nd^ treated MDD episodes was additionally determined and stratified by TRD and non-TRD episodes. Patient demographics included age, gender, geographic region, insurance type, and health plan type and were reported separately for those with 1^st^ treated MDD episodes and those with 2^nd^ treated MDD episodes.

### Statistical analyses

Descriptive statistics were utilized to describe patient demographics and measured outcomes. Mean and standard deviation (SD) were reported for continuous variables; percentages were reported for categorical variables. All statistical analyses were conducted using SAS Enterprise Guide 7 (SAS Institute Inc., Cary NC).

## Results

### Study population

**Fig 2:**
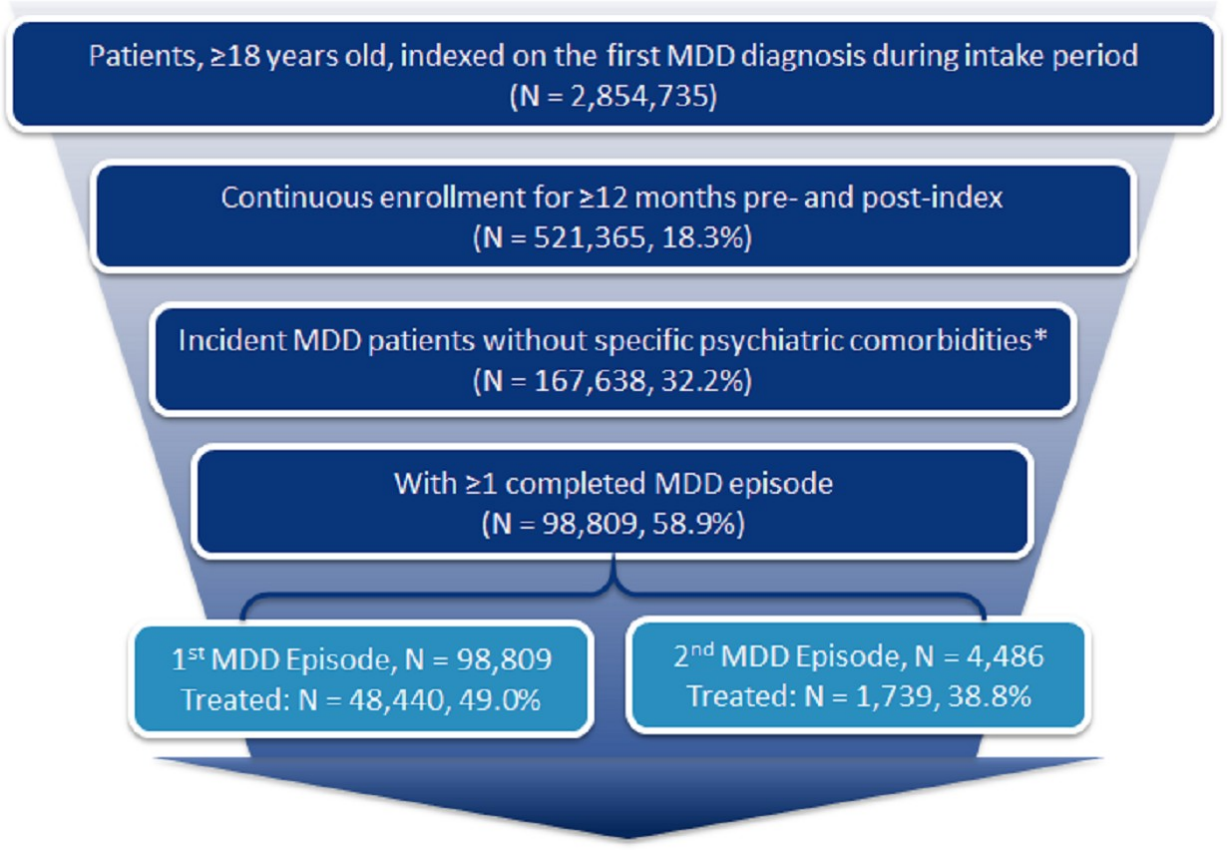
Attrition diagram. *Psychosis, schizophrenia, bipolar disorder, dementia and Tourette syndrome. MDD: major depressive disorder.

Patient attrition is shown in Fig 2. Of the 98,809 patients with ≥1 completed MDD episode during the study period, 49% (N = 48,440) were pharmacologically treated in the 1^st^ MDD episode. The mean age of patients with the 1^st^ treated MDD episode was 39.2 (SD: 15.4) years, 61.6% were female, and the majority had commercial insurance (94.9%) (S1 Table). Of the patients with ≥1 treated MDD episode, 3.5% (N = 1,739) had a 2^nd^ treated MDD episode (S1 Table) during the observed follow-up period. The mean age of patients with a 2^nd^ treated MDD episode was 38.6 (SD: 14.6) years and 67.3% were female (S1 Table).

### Characteristics of 1^st^ and 2^nd^ treated MDD episodes

**Fig 3:**
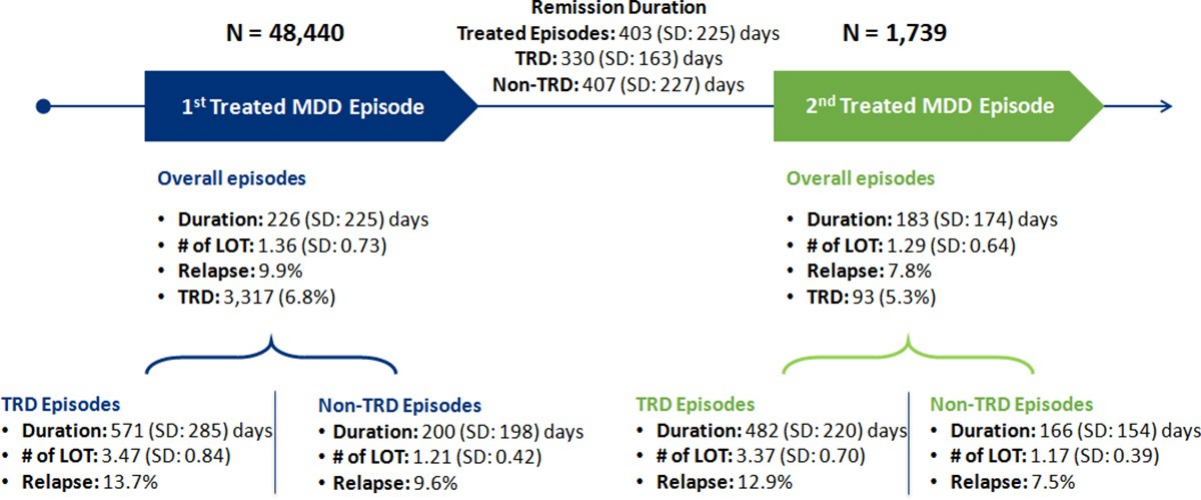
Characteristics of 1^st^ and 2^nd^ treated MDD episodes. LOT: line of therapy; MDD: major depressive disorder; TRD: treatment-resistant depression.

The characteristics of the 1^st^ and 2^nd^ treated MDD episodes are shown in Fig 3. The mean duration of 1^st^ treated MDD episodes was 226 (SD: 225) days, the mean number of AD/AP LOTs was 1.36 (SD: 0.73), and 9.9% involved relapse hospitalization during the episode. Of the 1^st^ MDD episodes, 6.8% (N = 3,317) of episodes were qualified as TRD. The mean duration of 1^st^ TRD episodes was 571 (SD: 285) days, the mean number of AD/AP LOTs was 3.47 (SD: 0.84), and 13.7% involved relapse hospitalization. The mean duration of 1^st^ non-TRD episodes was 200 (SD: 198) days, the mean number of AD/AP LOTs was 1.21 (SD: 0.42), and 9.6% involved relapse hospitalization.

The mean duration of 2^nd^ treated MDD was 183 (SD: 174) days, the mean number of AD/AP LOTs was 1.29 (SD: 0.64), and 7.8% involved relapse hospitalization during the episode. Of the 2^nd^ MDD episodes, 5.3% (N = 93) were considered as TRD. The mean duration of 2^nd^ TRD episodes was 482 (SD: 220) days, the mean number of AD/AP LOT was 3.37 (SD: 0.70), and 12.9% involved relapse hospitalization. The mean duration of 2^nd^ non-TRD MDD episodes was 166 (SD: 154) days, the mean number of AD/AP LOTs was 1.17 (SD: 0.39), and 7.5% involved relapse hospitalization.

The average remission time between 1^st^ and 2^nd^ treated MDD episodes was 403 (SD: 225) days; the average remission time for TRD episodes was shorter than that of non-TRD MDD episodes (330 [SD: 163] days vs. 407 [SD: 227] days). Among 1^st^ treated MDD episodes, the rate of recurrence was small. Among those with at least one-year follow-up after exiting a treatment episode, 4.3% had a subsequent treated episode. Among those with at least two-years follow-up, 7.2% had a subsequent treated episode.

### AD/AP treatment regimens during the 1^st^ treated MDD episode

**Fig 4:**
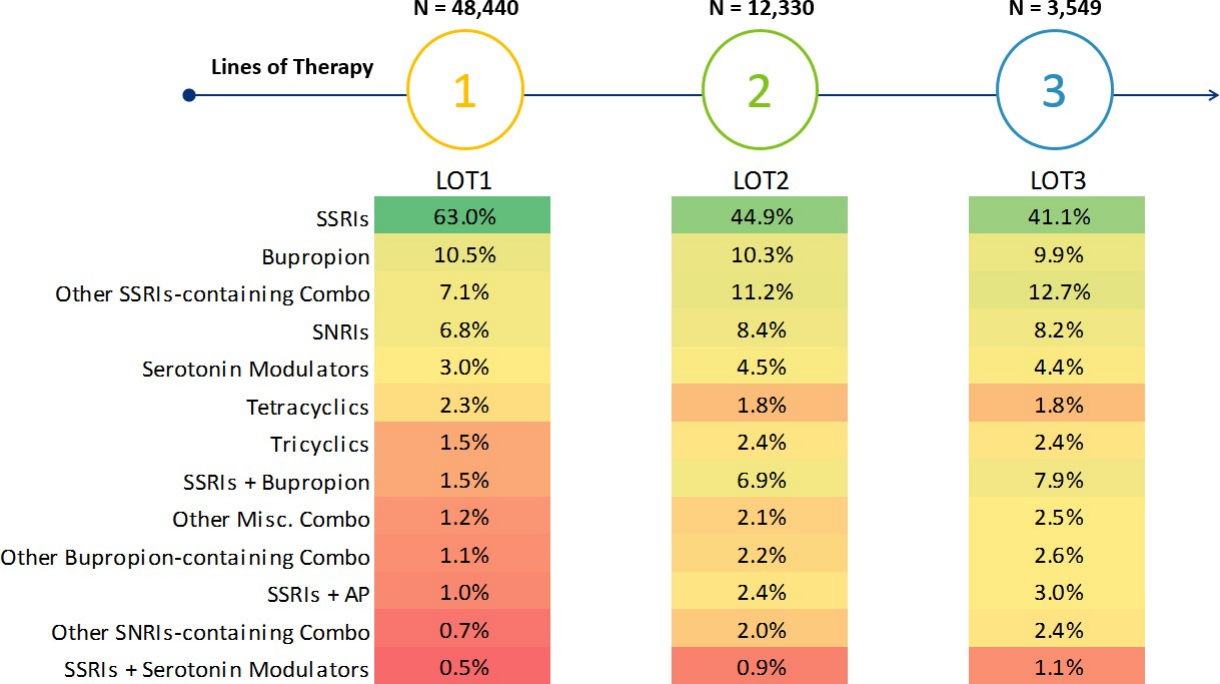
Patterns of antidepressant/antipsychotic treatment during the 1st treated MDD episode. AP: antipsychotic; Combo: combination; LOT: line of therapy; MDD: major depressive disorder Misc: miscellaneous; SNRIs: serotonin norepinephrine reuptake inhibitors; SSRIs: selective serotonin reuptake inhibitors.

Patterns of AD/AP treatment during the 1^st^ treated MDD episode are shown in Fig 4. Among 1^st^ treated MDD episodes (N = 48,440), 25.5% (N = 12,330) included a LOT2, and 7.3% (N = 3,549) included a LOT3. For LOT1, SSRI monotherapy was the predominant regimen (63.0%). Other AD regimens were used at a much lower frequency (bupropion: 10.5%; other SSRIs-containing combo: 7.1%; SNRIs: 6.8%). In LOT2 and LOT3, the plurality of regimens remained SSRI monotherapy (44.9% and 41.1%, respectively).

### Treatment sequence patterns during the 1^st^ treated MDD episode

Tables 1 and 2 show the treatment sequence patterns from LOT1 to LOT2 and from LOT2 to LOT3, respectively, during the 1^st^ treated MDD episode. Of the LOT1 treated with SSRIs, 24.6% (N = 7,519) involved a sequence to a LOT2, of which most remained on SSRI monotherapy (58.2%). For LOT1 treated with other AD/AP drug classes and drug combinations, the frequency of having a LOT2 was lowest with tricyclics (20.9%) and highest with SSRI + AP (34.4%). Generally, three regimens were disproportionately represented at LOT2: 1) SSRI monotherapy (left most column in Tables 1 and 2), 2) the same drug class used in LOT1 (diagonal cells in Tables 1 and 2), or 3) Other SSRI-containing Combo (the 10^th^ regimen column in Tables 1 and 2). Among LOT2 with a treatment sequence to LOT3, similar treatment patterns were observed as for LOT1 to LOT2.

**Table 1:** Treatment sequence patterns from LOT1 to LOT2 during the 1^st^ treated MDD episode.

LOT 1 Regimen Distribution			Began LOT 2		LOT 1 — > LOT 2 Matrix													
					LOT 2 Regimen													
					SSRIs	SNRIs	BPN	S Mod	Tetra-cyclics	Tri-cyclics	SSRI+BPN	SSRI+AP	SSRI+S Mod	Other SSRI-con-taining Comb	Other SNRI-con-taining Comb	Other BPN-con-taining Comb	Other Misc. Comb	Total
**Antidepressant Class**	N	%	n	%														
**SSRIs**	30,507	63.0%	7,519	24.6%	58.2%	6.4%	7.6%	2.8%	1.1%	1.6%	7.2%	2.5%	0.2%	10.6%	0.5%	0.4%	0.9%	**100%**
**SNRIs**	3,313	6.8%	846	25.5%	20.6%	39.4%	7.9%	5.1%	0.5%	2.7%	0.7%	0.7%	4.8%	5.9%	9.6%	0.5%	1.7%	**100%**
**BPN**	5,100	10.5%	1,264	24.8%	22.0%	5.6%	35.9%	2.6%	1.1%	2.0%	16.5%	0.2%	0.1%	2.3%	2.4%	8.6%	0.6%	**100%**
**S Mod**	1,435	3.0%	362	25.2%	25.4%	6.1%	3.9%	31.2%	1.9%	3.6%	0.8%	0.6%	3.3%	14.1%	0.0%	5.0%	4.1%	**100%**
**Tetracyclics**	1,091	2.3%	254	23.3%	20.9%	3.9%	5.1%	5.5%	28.0%	2.4%	0.0%	0.0%	1.2%	14.2%	2.8%	6.7%	9.4%	**100%**
**Tricyclics**	722	1.5%	151	20.9%	21.9%	6.0%	3.3%	7.3%	2.0%	39.7%	1.3%	0.0%	0.0%	7.3%	2.0%	2.6%	6.6%	**100%**
**SSRI + BPN**	706	1.5%	212	30.0%	32.5%	9.9%	7.1%	3.3%	1.9%	2.8%	14.2%	1.9%	0.0%	14.6%	2.4%	5.7%	3.8%	**100%**
**SSRs + AP**	483	1.0%	166	34.4%	22.9%	2.4%	3.0%	2.4%	2.4%	0.6%	3.6%	17.5%	0.0%	28.3%	5.4%	2.4%	9.0%	**100%**
**SSRI + S Mod**	234	0.5%	75	32.1%	16.0%	17.3%	4.0%	12.0%	2.7%	4.0%	0.0%	0.0%	10.7%	12.0%	13.3%	2.7%	5.3%	**100%**
**Other SSRI-containing Comb**	3,457	7.1%	1,067	30.9%	30.5%	4.2%	8.6%	7.3%	1.7%	2.6%	3.8%	3.8%	2.2%	26.0%	3.2%	2.3%	3.7%	**100%**
**Other SNRI-containing Comb**	322	0.7%	103	32.0%	14.6%	12.6%	8.7%	3.9%	0.0%	1.9%	3.9%	1.0%	7.8%	9.7%	22.3%	8.7%	4.9%	**100%**
**Other BPN-containing Comb**	509	1.1%	156	30.6%	19.9%	5.8%	13.5%	7.7%	0.6%	1.3%	6.4%	2.6%	1.9%	12.8%	5.1%	18.6%	3.8%	**100%**
**Other Misc. Comb**	561	1.2%	155	27.6%	21.9%	5.2%	4.5%	7.1%	2.6%	2.6%	0.0%	16.1%	1.3%	7.1%	4.5%	2.6%	24.5%	**100%**
**Grand Total**	**48,440**	**100.0%**	**12,330**		**5,533**	**1,037**	**1,273**	**553**	**218**	**297**	**849**	**302**	**117**	**1,377**	**251**	**268**	**255**	**12,330**

AP: antipsychotic; BPN: bupropion; Comb: combination; LOT: line of therapy; Misc: miscellaneous; S Mod: serotonin modulator; SNRI: serotonin norepinephrine reuptake inhibitor; SSRI: selective serotonin reuptake inhibitor

**Table 2:** Treatment sequence patterns from LOT2 to LOT3 during the 1^st^ treated MDD episode.

LOT 2 Regimen Distribution			Begin LOT 3		LOT 2 — > LOT 3 Matrix													
					LOT 3 Regimen													
					SSRIs	SNRIs	BPN	S Mod	Tetra-cyclics	Tri-cyclics	SSRI+BPN	SSRI+AP	SSRI+S Mod	Other SSRI-con-taining Comb	Other SNRI-con-taining Comb	Other BPN-con-aining Comb	Other Misc. Comb	Total
**SSRIs**	5,533	44.9%	1,460	26.4%	65.1%	5.5%	5.8%	3.4%	0.7%	1.4%	6.4%	1.7%	0.1%	8.6%	0.4%	0.2%	0.7%	**100%**
**SNRIs**	1,037	8.4%	290	28.0%	17.2%	42.4%	9.7%	4.5%	0.3%	3.1%	1.4%	0.0%	4.8%	2.8%	11.7%	0.3%	1.7%	**100%**
**BPN**	1,273	10.3%	351	27.6%	19.9%	6.8%	42.2%	2.6%	1.1%	1.1%	12.0%	0.3%	0.3%	1.7%	3.4%	8.0%	0.6%	**100%**
**S Mod**	553	4.5%	157	28.4%	17.2%	5.1%	8.9%	33.8%	3.8%	2.5%	0.6%	0.6%	4.5%	12.1%	0.0%	3.8%	7.0%	**100%**
**Tetracyclics**	218	1.8%	65	29.8%	12.3%	7.7%	1.5%	1.5%	41.5%	9.2%	0.0%	0.0%	0.0%	13.8%	1.5%	4.6%	6.2%	**100%**
**Tricyclics**	297	2.4%	85	28.6%	24.7%	4.7%	7.1%	10.6%	2.4%	30.6%	0.0%	0.0%	0.0%	16.5%	2.4%	0.0%	1.2%	**100%**
**SSRI + BPN**	849	6.9%	279	32.9%	28.3%	9.0%	8.6%	2.9%	0.4%	0.7%	22.2%	2.9%	0.7%	15.8%	3.9%	3.6%	1.1%	**100%**
**SSRI + AP**	302	2.4%	108	35.8%	16.7%	6.5%	4.6%	5.6%	0.0%	0.9%	3.7%	22.2%	0.9%	22.2%	5.6%	1.9%	9.3%	**100%**
**SSRI + S Mod**	117	0.9%	38	32.5%	18.4%	10.5%	5.3%	10.5%	0.0%	2.6%	0.0%	0.0%	15.8%	7.9%	21.1%	2.6%	5.3%	**100%**
**Other SSRI-containing Comb**	1,377	11.2%	450	32.7%	28.4%	8.0%	4.9%	7.8%	1.6%	2.9%	4.9%	4.2%	1.6%	23.3%	4.2%	3.1%	5.1%	**100%**
**Other SNRIs-containing Comb**	251	2.0%	85	33.9%	20.0%	8.2%	5.9%	4.7%	1.2%	0.0%	3.5%	1.2%	3.5%	7.1%	32.9%	5.9%	5.9%	**100%**
**Other BPN-containing Comb**	268	2.2%	92	34.3%	10.9%	6.5%	23.9%	3.3%	1.1%	2.2%	4.3%	1.1%	3.3%	13.0%	5.4%	20.7%	4.3%	**100%**
**Other Misc. Comb**	255	2.1%	89	34.9%	21.3%	7.9%	6.7%	2.2%	2.2%	3.4%	0.0%	9.0%	0.0%	9.0%	9.0%	6.7%	22.5%	**100%**
**Grand Total**	**12,330**	**97.9%**	**3,549**		**1,404**	**337**	**368**	**196**	**62**	**91**	**236**	**88**	**45**	**384**	**140**	**98**	**100**	**3,549**

AP: antipsychotic; BPN: bupropion; Comb: combination; LOT: line of therapy; Misc: miscellaneous; S Mod: serotonin modulator; SNRI: serotonin norepinephrine reuptake inhibitor; SSRI: selective serotonin reuptake inhibitor

### Most common treatment patterns (LOT1 to LOT3) for 1^st^ TRD episodes

The 20 most commonly observed treatment sequence patterns, which comprised 1,743 (52.5%) of the total 3,317 TRD episodes, are shown in Table 3. By far the most frequently observed treatment pattern of 1^st^ TRD episodes was multiple SSRIs in different LOTs, with 23.9% treated with SSRIs in LOT1, LOT2, and LOT3. All other treatment patterns were present in <5% of patients.

**Table 3:** Top 20 treatment patterns (LOT 1 to LOT 3) for 1^st^ treated TRD episodes.

	N	%
Total 1^st^ treated TRD episodes (≥3 LOTs)	3,317	100%
SSRIs—> SSRIs—> SSRIs	792	23.9%
SSRIs—> Other SSRIs-containing Combo—> SSRIs	81	2.4%
SSRIs—> SSRIs—> Other SSRIs-containing Combo	79	2.4%
Bupropion—> Bupropion—> Bupropion	78	2.4%
SSRIs—> SSRIs—> SSRIs + Bupropion	70	2.1%
SSRIs—> SSRIs—> Bupropion	66	2.0%
SSRIs—> SSRIs—> SNRIs	62	1.9%
SNRIs—> SNRIs—> SNRIs	56	1.7%
Other SSRIs-containing Combo—> SSRIs—> SSRIs	51	1.5%
SSRIs—> SSRIs + Bupropion—> SSRIs	49	1.5%
SSRIs—> Bupropion—> SSRIs	48	1.4%
SSRIs—> Other SSRIs-containing Combo—> Other SSRIs-containing Combo	44	1.3%
SSRIs—> SNRIs—> SNRIs	42	1.3%
SSRIs—> Bupropion—> Bupropion	39	1.2%
Bupropion—> SSRIs—> SSRIs	37	1.1%
SSRIs—> SNRIs—> SSRIs	33	1.0%
SSRIs—> SSRIs—> Serotonin Modulators	31	0.9%
SSRIs—> SSRIs + Bupropion—> SSRIs + Bupropion	31	0.9%
Other SSRIs-containing Combo—> Other SSRIs-containing Combo—> Other SSRIs-containing Combo	29	0.9%
SSRIs—> Bupropion—> SSRIs + Bupropion	25	0.8%
**TRD episodes comprising 20 most common treatment patterns**	**1,743**	**52.5%**

Combo: combination; LOT: line of therapy; SNRIs: serotonin norepinephrine reuptake inhibitors; SSRIs: selective serotonin reuptake inhibitors

## Discussion

In recent years there has been considerable attention given to TRD in the published literature. However, information on MDD and TRD at the episode level is lacking with respect to the number and types of AD/AP regimens used as well as the sequential pattern of LOT transitioning. A notable finding of our study was that less than half of MDD patients were pharmacologically treated; and the MDD episodes in general were lengthy, lasting over 220 days. In addition, TRD episodes were nearly three times longer than non-TRD MDD episodes for both 1^st^ and 2^nd^ treated episodes. Despite pharmacologic treatment, 10% of MDD episodes involved a relapse resulting in hospitalization. Despite use of an average 3.5 LOTs during 1^st^ TRD episodes, TRD episodes were associated with more relapse hospitalizations than non-TRD MDD episodes. The durations of MDD/TRD episodes, number of relapse hospitalizations, and frequency of changing LOTs observed in this study highlight the significant unmet need for alternative novel and/or supplemental treatment options to the conventional spectrum of ADs used for the management of MDD and TRD.

Approximately two-thirds of 1^st^ treated MDD episodes observed in this study were treated with SSRIs during LOT1. SSRIs remained the most common treatment used in LOT2 and LOT3 during the 1^st^ MDD and TRD episodes. Moreover, many patients with TRD cycled within the SSRI class. This preference for SSRIs may be reflective of clinician or patient familiarity, affordability, or the perception that all classes of oral antidepressants offer similar efficacy, but SSRIs offer better tolerability compared with other classes. These findings suggest a need for new antidepressants with improved efficacy for patients with TRD. Adjunctive APs do offer incremental efficacy compared with continued oral antidepressant monotherapy. Nevertheless, APs were used infrequently in the 1^st^ MDD episode, and the highest fraction of patients receiving SSRIs + AP in LOT1 transitioned to LOT2. These two findings may reflect the tolerability burden of adjunctive AP. When interpreting these results, it is important to recognize that starting and stopping the same medication after the requisite gap counts as a new LOT.

The recurrence rate of MDD episodes could not be explicitly determined in this study. Due the variable length follow-up, it was not possible to interpret the number of 2^nd^ treated MDD episodes compared with the number of 1^st^ treated MDD episodes as a rate of progression, since patients may not have had long enough follow-up time to detect a 2^nd^ episode. Nevertheless, we observed that among those who completed an episode with sufficient post-episode follow-up, the recurrence risk was low and appeared to decline over time. That is, the longer a patient remained out of a treatment episode the lower the chance of episode recurrence. This relatively low percentage should be interpreted in the context of the 180-day clean period that was used in this study. Although further study is needed, these results suggest that patients who achieve episode remission for 6 months have a good prognosis; this could become an important clinical goal. Given that the MDD episodes are typically long in duration, it is critical that patients receive better and perhaps more intensive treatment and care management because MDD, and especially TRD, are clinically debilitating and worsened dramatically by the increased likelihood for suicide. In a recent study, Bauer et al conducted an analysis of large-scale clinical trials, and concluded that algorithm-guided treatment for MDD, in which systematic assessment of treatment response is performed at critical decision points, provides a structured mechanism that is associated with improved treatment outcomes versus usual treatment [16]. Alongside the refractory nature of MDD, especially TRD, findings from this study emphasize the significant need for more widespread use of improved treatment strategies for patients diagnosed with MDD.

Due to the nature of claims database studies, we were not able to directly assess other clinical measures of treatment responsiveness (e.g., diminution of symptoms) of patients with MDD. Instead proxy measures (e.g., prescription fills, subsequent MDD diagnoses) were used to indirectly detect treatment responsiveness and changes in treatment. For this analysis of patients newly diagnosed with MDD, we used a restrictive 365-day pre-index “clean period” during which no MDD diagnosis or prescription fill for AD/AP was identified. Additionally, we required patients to have a ≥180-day clean period between MDD episodes to define a completed episode and entry into remission. These design aspects, while restrictive, were rigorous and conservative in the identification of potential treatment non-response. However, they might have contributed to a TRD prevalence estimate that is lower than that reported in previous studies [13,14].

This was an episode-level retrospective cohort study that utilized claims data extracted from the MarketScan databases. It has limitations that should be recognized when interpreting these results. Among these are that administrative claims data are collected for facilitating payment for healthcare services and not for research. When claims data are used to identify diagnoses, results may be incomplete or inaccurate, leading to potential misclassification bias. Also, generic prescriptions paid out-of-pocket may not be captured in claims databases. This may have led to an underestimate of both drug utilization and MDD episode duration. Claims for prescription fills may not necessarily be reflective of the actual medication taken. This study utilized an empirical clean period length and maximum permissible gap, which may have impacted the identification of MDD episodes and LOTs. Also, this study only included patients covered by commercial or Medicare supplemental insurance; therefore, the results may not be generalizable to other populations with other types of insurance coverage (i.e., Medicaid). By study design, only patients with completed treatment episodes were included in the study population. Thus, these study results may not generalize well to patients who have extended treated MDD episodes.

## Conclusions

This study utilized an episodic approach for evaluating the treatment journey of patients with newly diagnosed MDD. The results suggest that, compared with non-TRD MDD episodes, TRD episodes are longer, more frequently involve relapse hospitalization and have shorter duration of remission. This study also reveals a real-world treatment pattern of AD during the treated MDD episode, in which the most common AD drug class used in sequential LOTs was the same one used in the initial LOT. Of potential AD treatment classes, an SSRI was the most frequently used treatment across LOTs. Findings from this study may help to better understand the disease burden of TRD and unmet treatment needs in the management of patients with MDD, especially those with TRD.

## Supporting information

S1 Table Patient Demographics. CDHP: consumer-driven health plan; HMO: health maintenance organization; MDD: major depressive disorder; POS: point-of-service plan; PPO: preferred provider organization.
(DOCX)

## References

[1] National Institute of Mental Health. November 2017. Bethesda, MD. Available from: https://www.nimh.nih.gov/health/statistics/major-depression.shtml

[2] Murray CJ, Atkinson C, Bhalla K, Birbeck G, Burstein R, Chou D, et al The state of U.S. health, 1990–2010: burden of diseases, injuries and risk factors. JAMA. 2013;310: 591–608. 10.1001/jama.2013.13805 23842577 PMC5436627

[3] Greenberg PE, Fournier AA, Sisitsky T, Pike CT, Kessler RC. The economic burden of adults with major depressive disorder in the United States (2005 and 2010). J Clin Psychiatry. 2015;76: 155–162. 10.4088/JCP.14m09298 25742202

[4] Rush AJ, Trivedi MH, Wisniewski SR, Nierenberg AA, Stewart JW, Warden D, et al Acute and longer-term outcomes in depressed outpatients requiring one or several treatment steps: a STAR*D report. Am J Psychiatry. 2006;163: 1905–1917. 10.1176/ajp.2006.163.11.1905 17074942

[5] Nemeroff CB. Prevalence and management of treatment-resistant depression. J Clin Psychiatry. 2007;68: 17–25. 17640154

[6] Gaynes BN, Asher G, Gartlehner AG, Hoffman V, Green J, Boland J, et al Definition of treatment-resistant depression in the Medicare population. Agency for Healthcare Research and Quality 2 2018 Rockville, MD Available from: https://www.ncbi.nlm.nih.gov/books/NBK526366/30260611

[7] Vergunst FK, Fekadu A, Wooderson SC, Tunnard CS, Rane LJ, Markopoulou K, et al Longitudinal course of symptom severity and fluctuation in patients with treatment-resistant unipolar and bipolar depression. Psychiatry Res. 2013;207: 143–149. 10.1016/j.psychres.2013.03.022 23601791

[8] Coryell W, Young EA. Clinical predictors of suicide in primary major depressive disorder. J Clin Psychiatry. 2005;66: 412–417. 15816781 10.4088/jcp.v66n0401

[9] Crown WH, Finkelstein S, Berndt ER, Ling D, Poret AW, Rush AJ, et al The impact of treatment-resistant depression on health care utilization and costs. J Clin Psychiatry. 2002;63: 963–971. 12444808 10.4088/jcp.v63n1102

[10] Johnston KM, Powell LC, Anderson IM, Szabo S, Cline S. The burden of treatment-resistant depression: A systematic review of the economic and quality of life literature. J Affect Disord. 2019;242: 195–210. 10.1016/j.jad.2018.06.045 30195173

[11] Mrazek DA, Hornberger JC, Altar CA, Degtia I. A review of the clinical, economic, and societal burden of treatment-resistant depression: 1996–2013. Psychiatr Serv. 2014;65: 977–987. 10.1176/appi.ps.201300059 24789696

[12] Cuijpers P, Smit F. Excess mortality in depression: a meta-analysis of community studies. J Affect Disord. 2002;2: 227–236. 10.1016/s0165-0327(01)00413-x 12450639

[13] Kubitz N, Mehra M, Potlure RC, Garg N, Crossrow. Characterization of treatment resistant depression episodes in a cohort of patients from a US commercial claims database. PLoS One. 2013;8: e76882 10.1371/journal.pone.0076882 24204694 PMC3799999

[14] Amos TB, Tandon N, Lefebvre P, Pilon D, Kamstra RL, Pivneva I, et al Direct and indirect cost burden and change of employment status in treatment-resistant depression: a matched-cohort study using a US commercial claims database. J Clin Psychiatry. 2018;79 pii: 17m11725. 10.4088/JCP.17m11725 29474009

[15] Olfson M, Amos TB, Benson C, McRae J, Marcus SC. Prospective service use and health care costs of Medicaid beneficiaries with treatment-resistant depression. J Manag Care Pharm. 2018;24: 226–236. 10.18553/jmcp.2018.24.3.226 PMC10398231 29485948

[16] Bauer M, Rush AJ, Ricken R, Pilhatsch M, Adli M. Algorithms for treatment of major depressive disorder: efficacy and cost-effectiveness. Pharmacopsychiatry. 20198;52: 117–125. 10.1055/a-0643-4830 29986372

