# Supplementary material for: An episode level evaluation of the treatment journey of patients with major depressive disorder and treatment-resistant depression
Source: PLoS One. 2019 Aug 8;14(8):e0220763. doi: 10.1371/journal.pone.0220763 (PMC6687173; doi:10.1371/journal.pone.0220763)
Supplement: S1 Table — CDHP: consumer-driven health plan; HMO: health maintenance organization; MDD: major depressive disorder; POS: point-of-service plan; PPO: preferred provider organization. (DOCX) [file pone.0220763.s001.docx]

|  | **1^st^ Treated MDD Episode** | **2^nd^ Treated MDD Episode** |
| --- | --- | --- |
| **Number of Episodes** | **48,440** | **1,739** |
| **Age, Mean (SD)** | 39.2 (15.4) | 38.6 (14.6) |
| **Female, n (%)** | 29,837 (61.6%) | 1,171 (67.3%) |
| **Region, n (%)** |  |  |
| Northeast | 6,821 (14.1%) | 247 (14.2%) |
| North Central | 10,488 (21.7%) | 408 (23.5%) |
| South | 15,436 (31.9%) | 555 (31.9%) |
| West | 15,177 (31.3%) | 514 (29.6%) |
| Unknown | 518 (1.1%) | 15 (0.9%) |
| **Insurance type, n (%)** |  |  |
| Commercial | 45,989 (94.9%) | 1,656 (95.2%) |
| Medicare | 2,451 (5.1%) | 83 (4.8%) |
| **Health plan type, n (%)** |  |  |
| Comprehensive | 2,042 (4.2%) | 90 (5.2%) |
| HMO | 10,541 (21.8%) | 398 (22.9%) |
| PPO | 26,478 (54.7%) | 931 (53.5%) |
| POS | 4,310 (8.9%) | 204 (11.7%) |
| CDHP | 4,772 (9.9%) | 110 (6.3%) |
| Others | 297 (0.6%) | 6 (0.3%) |
